# Supplementary material for: PBMC fixation and processing for Chromium single-cell RNA sequencing
Source: J Transl Med. 2018 Jul 17;16:198. doi: 10.1186/s12967-018-1578-4 (PMC6050658; doi:10.1186/s12967-018-1578-4)
Supplement: Supplementary file 3 — Additional file 3: Figure S1. Resuspension in ×3 SSC preserved cell RNA integrity. a. The methanol-fixed PBMCs resuspended in ×3 or ×5 SSC buffer showed high quality of RNA determined with Bioanalyzer traces. b. The new processing method was validated in several other cell types resuspended in ×3 SSC for 30 min. The RIN numbers were significantly improved in primary cell types and cell lines with a p value of 0.00001 and 0.008 respectively. [file 12967_2018_1578_MOESM3_ESM.pptx]

## Slide 1
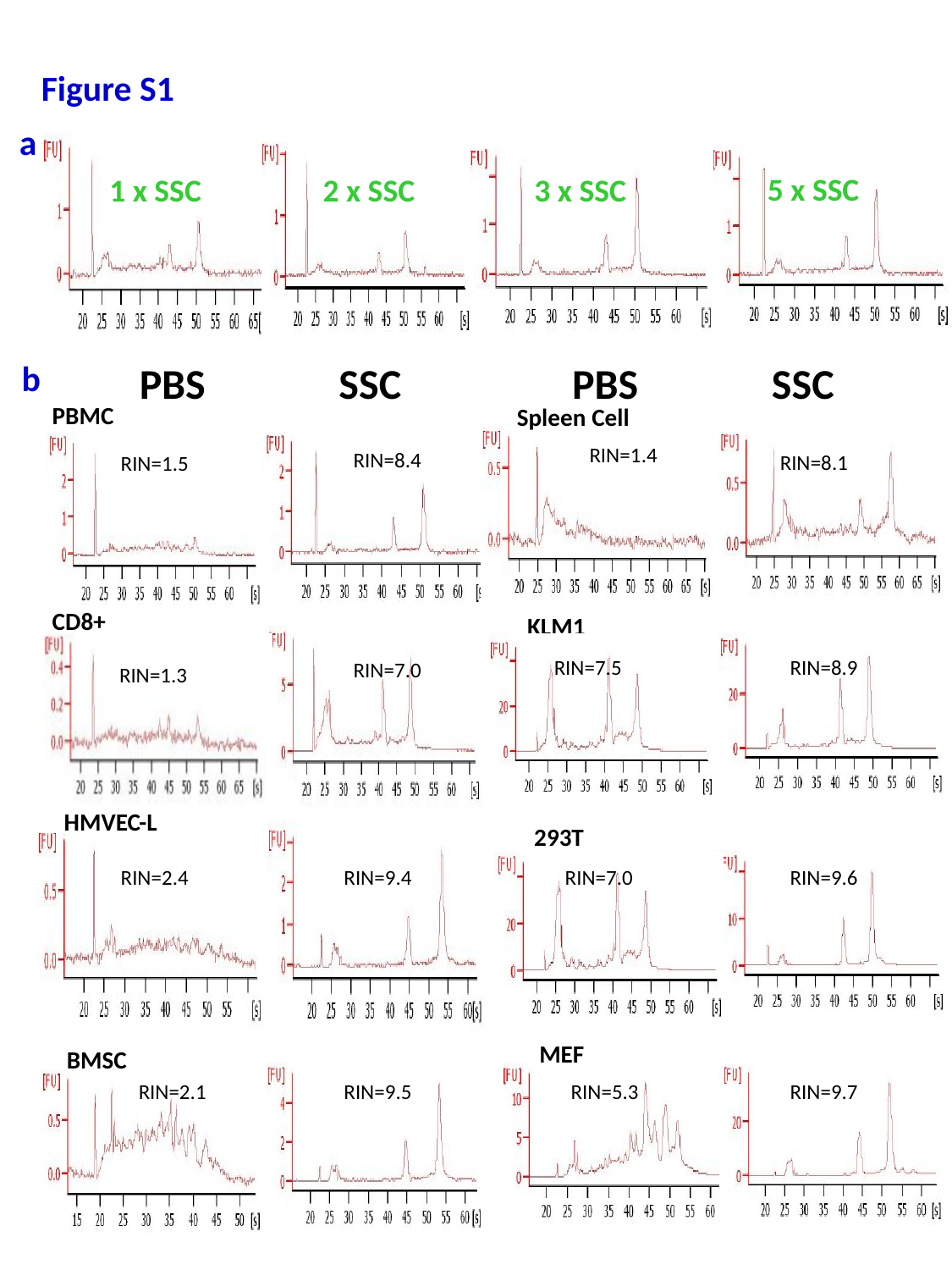

Figure S1
a
5 x SSC
1 x SSC
3 x SSC
2 x SSC
b
PBS SSC
PBS SSC
PBMC
Spleen Cell
RIN=1.4
RIN=8.4
RIN=8.1
RIN=1.5
CD8+
KLM1
RIN=8.9
RIN=7.5
RIN=7.0
RIN=1.3
HMVEC-L
293T
RIN=9.4
RIN=7.0
RIN=2.4
RIN=9.6
MEF
BMSC
RIN=2.1
RIN=9.5
RIN=5.3
RIN=9.7
