# Supplementary material for: PBMC fixation and processing for Chromium single-cell RNA sequencing
Source: J Transl Med. 2018 Jul 17;16:198. doi: 10.1186/s12967-018-1578-4 (PMC6050658; doi:10.1186/s12967-018-1578-4)
Supplement: Supplementary file 4 — Additional file 4: Figure S2. Fixation processing did not aggregate the cells. PBMCs with greater than 2500 genes detected (a) or CD8+ cells with more than 2000 genes detected (b) were flagged because these droplets usually contain more than one cell (doublet or multiplet). Fixation did not increase the rate of these GEMs/cells. Methanol-fixed PBMCs remained microscopically visible as single, intact round cells with the similar size as live ones. [file 12967_2018_1578_MOESM4_ESM.pptx]

## Slide 1
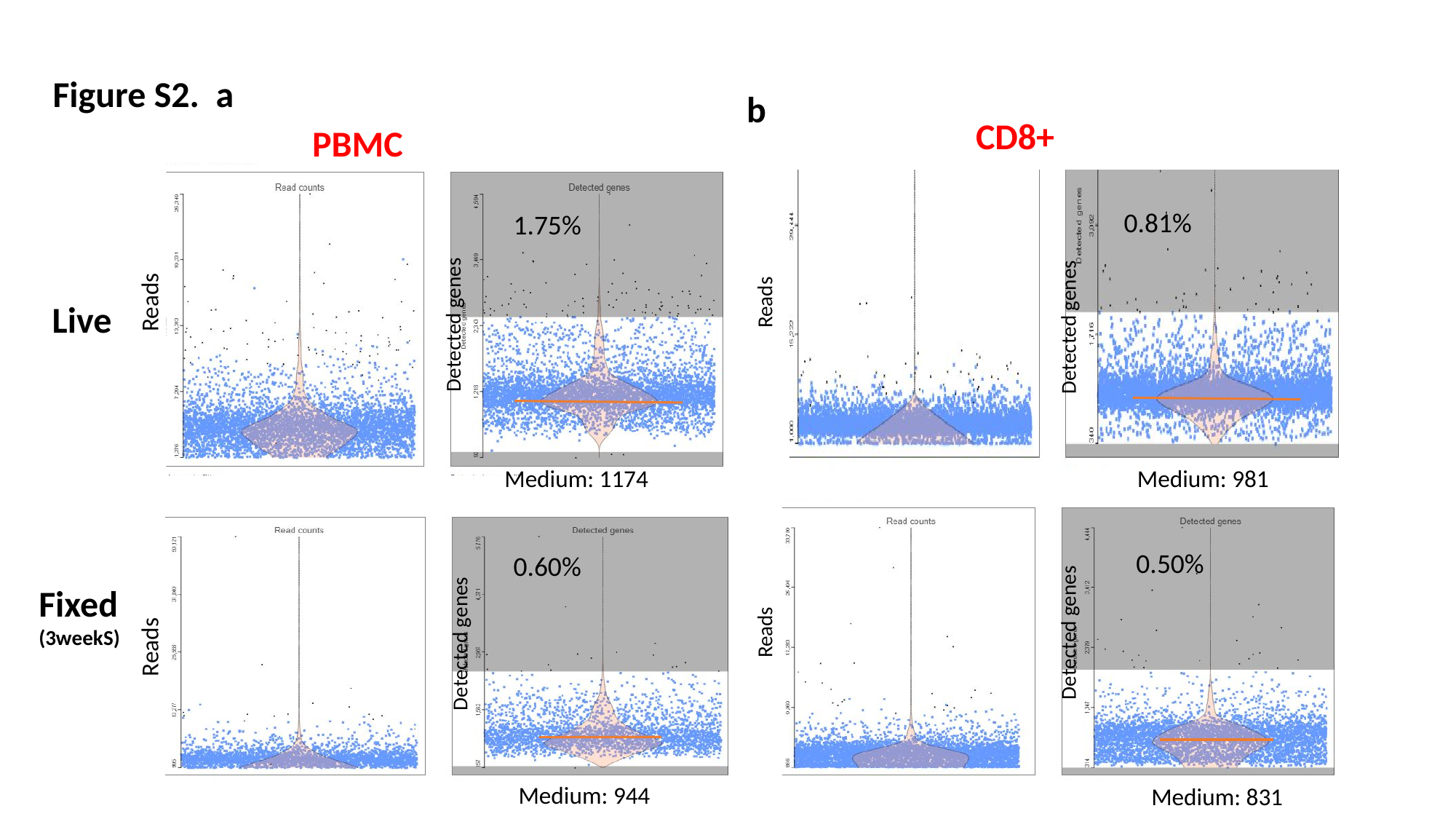

Figure S2. a
b
CD8+
PBMC
0.81%
1.75%
Detected genes
Detected genes
Reads
Reads
Live
Medium: 1174
Medium: 981
0.50%
0.60%
Detected genes
Fixed
(3weekS)
Detected genes
Reads
Reads
Medium: 944
Medium: 831
