# Supplementary material for: PBMC fixation and processing for Chromium single-cell RNA sequencing
Source: J Transl Med. 2018 Jul 17;16:198. doi: 10.1186/s12967-018-1578-4 (PMC6050658; doi:10.1186/s12967-018-1578-4)
Supplement: Supplementary file 6 — Additional file 6: Figure S3. tSNE projection of live and fixed PBMCs from donor DTM-X (a) and donor (b). Cells were grouped using graph-based method. Classification of PBMCs was inferred from the annotation of cluster-specific genes, and based on expression of some well-known markers of immune cell types. Although fixation lead to changes of the relative distances of the clusters due to the loss of genes detected, it did not impact the resolution of the low abundant populations (B, NK, DC) in each sample. Subpopulations were detected from fixed PBMCs at a similar proportion to those of live PBMCs (Table 1). [file 12967_2018_1578_MOESM6_ESM.pptx]

## Slide 1
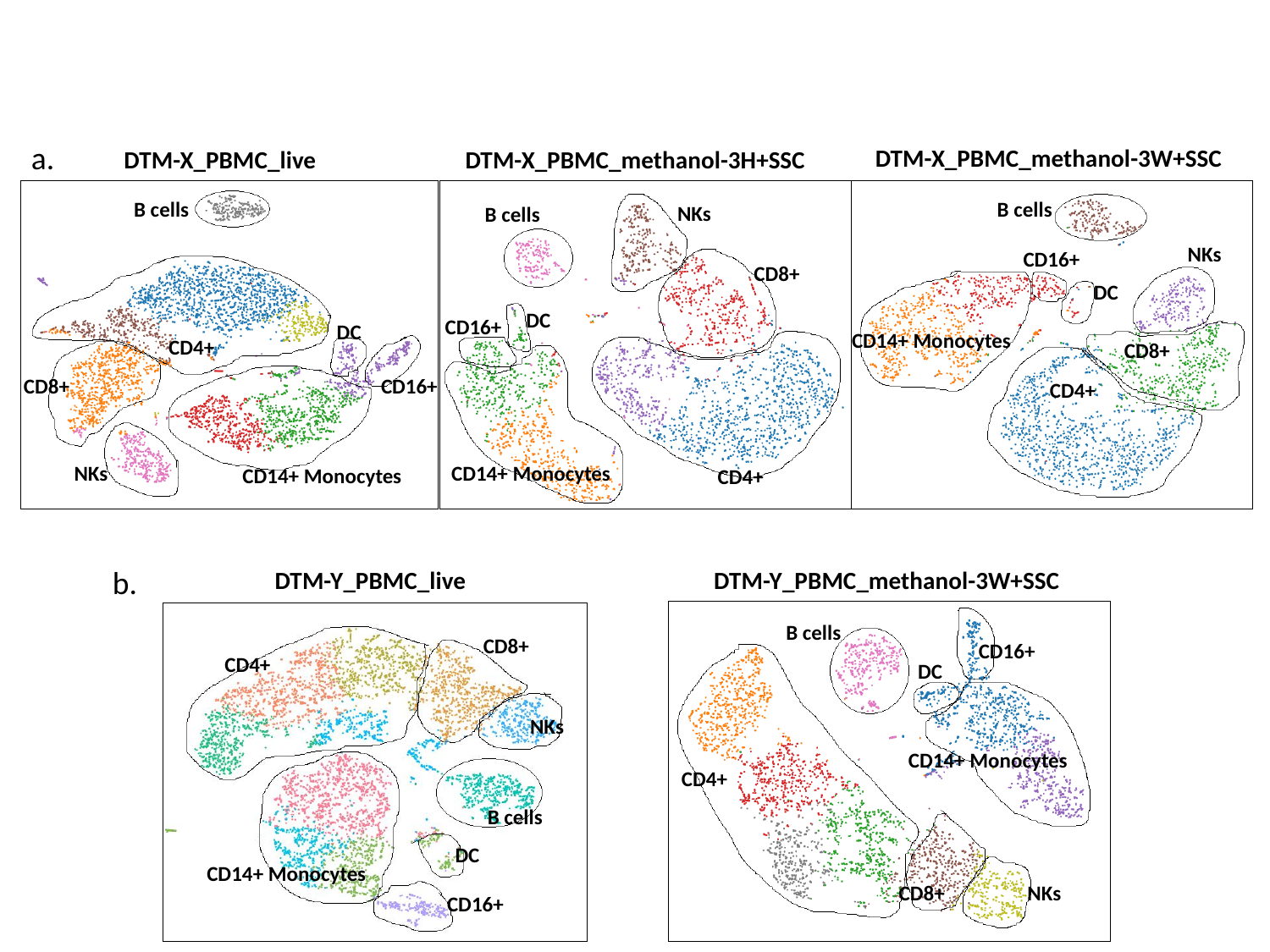

a.
DTM-X_PBMC_methanol-3W+SSC
DTM-X_PBMC_methanol-3H+SSC
DTM-X_PBMC_live
B cells
B cells
NKs
B cells
NKs
CD16+
CD8+
DC
DC
CD16+
DC
CD14+ Monocytes
CD4+
CD8+
CD16+
CD8+
CD4+
NKs
CD14+ Monocytes
CD14+ Monocytes
CD4+
b.
DTM-Y_PBMC_methanol-3W+SSC
DTM-Y_PBMC_live
B cells
CD8+
CD16+
CD4+
DC
NKs
CD14+ Monocytes
CD4+
B cells
DC
CD14+ Monocytes
CD8+
NKs
CD16+
